# Against the grain: Leveraging machine learning to analyze mudbrick structures

**DOI:** 10.1371/journal.pone.0349295

**Published:** 2026-05-21

**Authors:** Sofia Kouki, Marta Lorenzon, Benjamín Cutillas-Victoria, Achim Lichtenberger, Torben Schreiber, Mkrtich Zardaryan, Anno Hein, Georgios Polymeris

**Affiliations:** 1 Interdisciplinary Center for Archaeology and the Evolution of Human Behavior, University of Algarve, Faro, Portugal; 2 Department of Cultures, Faculty of Arts, University of Helsinki, Helsinki, Finland; 3 Centre of Excellence in Ancient Near Eastern Empires, University of Helsinki, Helsinki, Finland; 4 Grupo de Investigación en Arqueología Prehistórica, Universidad Complutense de Madrid, Madrid, Spain; 5 Institut für Klassische Archäologie und Christliche Archäologie, Archäologisches Museum, Universität Münster, Münster, Germany; 6 Institute of Archaeology and Ethnography, National Academy of Sciences of the Republic of Armenia, Yerevan, Armenia; 7 Institute of Nanoscience and Nanotechnology, NCSR “Demokritos”, Athens, Greece; Universidad de Sevilla, SPAIN

## Abstract

Mudbricks have been a fundamental building material since the Neolithic, yet their compositional variability and technological flexibility can be challenging for systematic and reproducible fabric characterization. Morphometric parameters such as grain size and shape influence the physical properties of mudbricks, providing insights into raw material selection, preparation practices, and construction techniques. While petrographic point counting remains the standard approach for fabric assessment, it is time-consuming, subjective, and difficult to standardize across studies. This study evaluates the application of automated image analysis for quantitative grain morphometry in petrographic thin sections of archaeological mudbricks. We developed a computational workflow based on K-means clustering to extract grain size, sorting, and shape descriptors from 45 cross-polarized light images from two geographically and chronologically distinct sites: the Urartian and Hellenistic city of Artaxata (Armenia) and the Early Iron Age hillfort of Los Villares de la Encarnación (Spain), both previously characterized through detailed petrographic analysis. We assessed the inter-site grain morphometric differences using non-parametric statistical testing, multivariate ordination, and supervised classification, and the automated results were compared with existing fabric descriptors. Our results indicate statistically significant differences in grain size, sorting, and shape between the two sites, with clear separation in multivariate space and good classification accuracy (>84%). Automated morphometric patterns show strong correspondence with the previously defined petrographic fabrics, indicating that automated grain morphometry captures assemblage-level technological variability. The proposed workflow establishes a methodological foundation for quantitative and comparative studies in earthen building architecture and supports the integration of computational tools into archaeological thin-section petrography.

## Introduction

Mudbrick has served as a construction material since the Neolithic period, offering an accessible and simple option [1,2] employed up to the present day and across five continents. Mudbrick constructions are typically made by mixing clay with varying ratios of silt, vegetal temper, and water, which are then shaped into blocks that solidify through sun drying [3]. This straightforward process results in a versatile construction material suitable for a variety of diverse structures, ranging from small dwellings and auxiliary domestic structures to palaces and fortifications [4–6].

The properties of these earthen building materials (EBM onwards) are significantly influenced by the characteristics of the selected soils [7]. The composition depends on the naturally present aplastic inclusions and, in some cases, human-selected aggregates, including form, angularity, texture, and the fine-to-coarse ratio. These factors affect the workability, durability, and overall strength of the material. Understanding the influence of these characteristics allows archaeologists to gain insights into several aspects of reconstructing the earthen building architecture, including the raw material selection and the manufacturing process [1,8–11].

Fabric analysis on EBM, specifically grain size and shape analysis, is an extension of the ceramic petrographic studies [11–14] that offer numerous advantages when complemented with micromorphology. This adaptation has opened up new approaches to the investigation of material culture, shedding light on architectural patterns, environmental interaction, labour organization, or identity formation in different geographical scenarios and chronologies [15–17].

Specifically in mudbrick construction, the ratio of clay, silt, and sand is a crucial parameter that often defines the composition and quality of the building materials used. The repetition in similar ratios may indicate a consistency of recipes over time within a single group, such as household production and/or craftspeople group, or a single manufacturing event that occurred at a specific time [8,16,18]. As mudbrick recipes can be quite flexible, including a percentage of sand that ranges between 15% to 70% with an appropriate degreaser, particle size analysis has been considered one of the most relevant variables to assess compositional homogeneity, as it reflects not only the environmentality of each geographical area but also specific selection choices made by the mudbrick maker [15,16].

Although widely applied, traditional observation methods such as point counting in grain size analysis are subject to limitations, including extensive time requirements, high subjectivity, dependence on user expertise, and lack of reproducibility in results [19–21]. In response to these constraints, digital image analysis and machine learning algorithms applied to other kinds of archaeological materials provide a promising alternative, producing more efficient, quick, and objective results [10,22–28]. However, its suitability has not been tested on construction materials, neither on EBM nor on others, despite the value that the different inclusions and aggregates have for the structure of the manufactured material itself.

**Fig 1:**
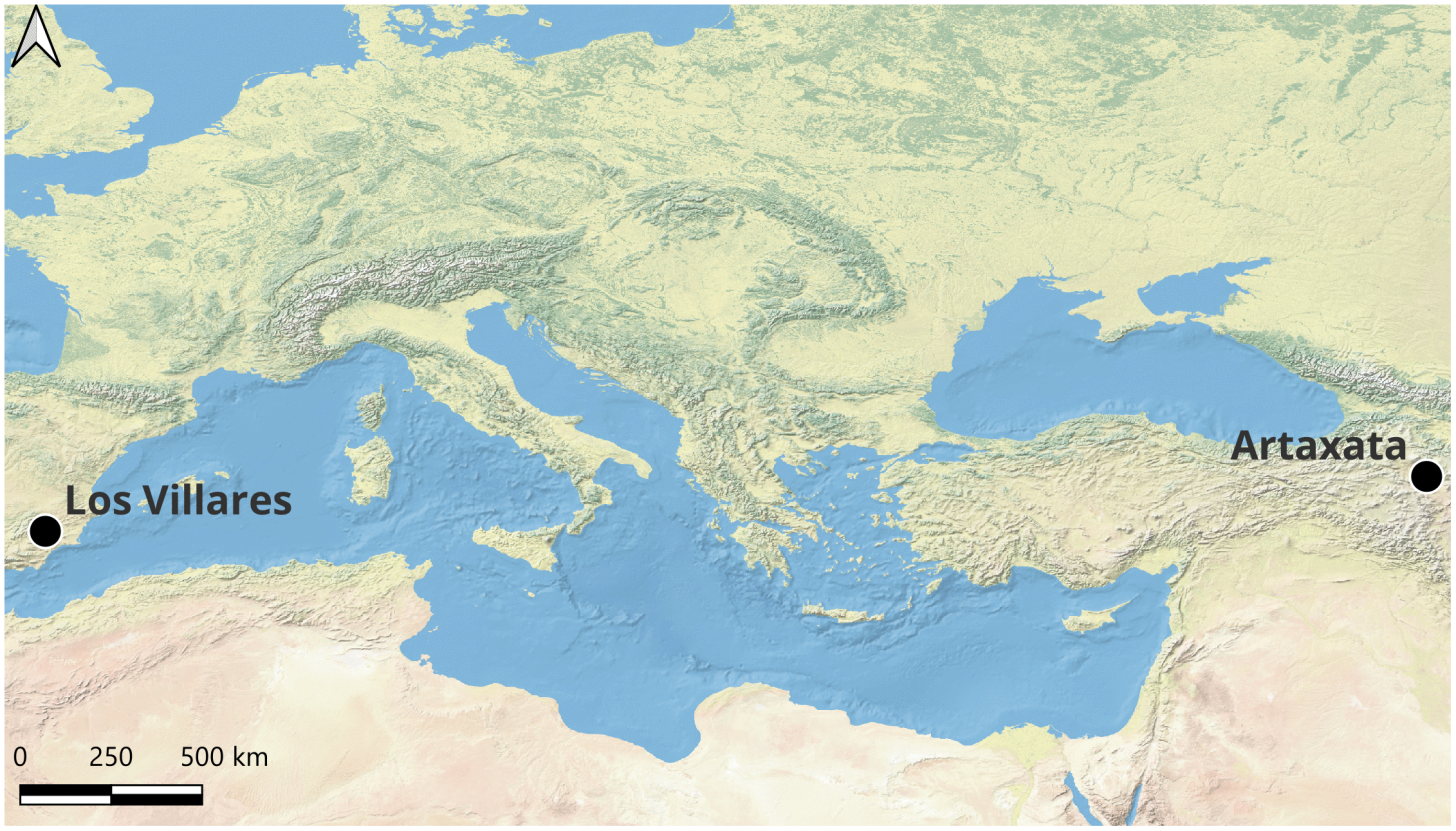
Study site locations. Map showing the positioning of Los Villares de la Encarnación and Artaxata**.** Basemap: Digital Elevation Model (DEM) data sourced from Natural Earth (public domain; https://www.naturalearthdata.com/). Map created using QGIS 4.0.1.

This study develops and validates an automated workflow for quantitative grain morphometry in earthen building materials thin sections from two geographically and temporally distinct sites (Fig 1): the Urartian and Hellenistic city of Artaxata (Ararat region, Armenia) and the Early Iron Age hillfort of Los Villares de la Encarnación (Caravaca de la Cruz, Spain). Both assemblages were previously analyzed through comprehensive geoarchaeological studies of earthen architecture and natural resources [29,30]. These sites, characterized by distinct geological contexts and fabric compositions, provide an ideal test case for assessing whether automated morphometric analysis can reliably distinguish between different production traditions linked with the manufacture of mudbricks.

Our study has three objectives: (1) to develop and document a reproducible computational workflow for automated segmentation and quantitative morphometric analysis of aplastic inclusions in EBM thin sections, following established petrographic standards; (2) to apply this workflow to quantitatively characterize and compare mudbrick fabrics from two geographically and chronologically distinct archaeological sites, Artaxata and Los Villares de la Encarnación, using grain size, sorting, and shape parameters; and (3) to evaluate whether quantitative grain morphometry captures archaeologically meaningful differences between assemblages from different archaeological contexts, thereby assessing its potential as a comparative tool for identifying technological consistency and variability in earthen architecture.

By integrating automated image analysis with statistical validation, this study also evaluates the potential of quantitative grain morphometry to provide an objective and reproducible framework for fabric characterization that complements traditional petrographic analysis.

## Archaeological background

### Artashat-Artaxata (Armenia)

**Fig 2:**
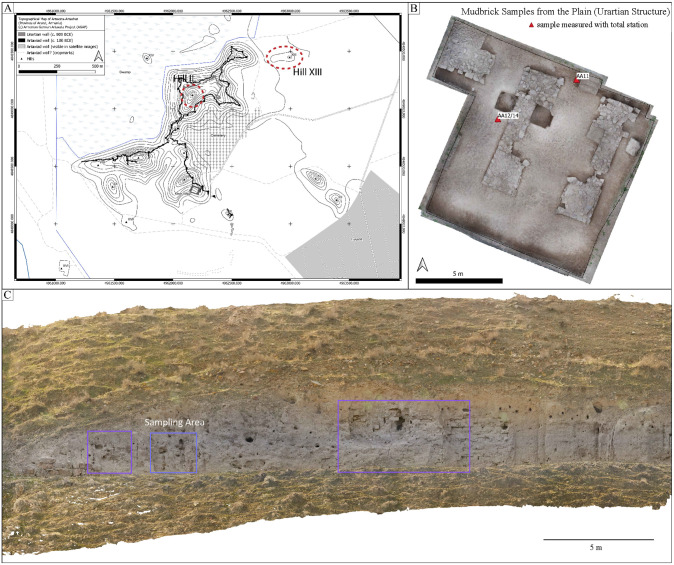
Photographs of the sites of Artaxata. **A)** Plan of the Artaxata site, with its hills and fortification system. **B)** and **C)** Location of the sampling areas within specific structures of Artaxata (Reprinted from [30] under CC BY 4.0 license).

The Hellenistic city of Artashat-Artaxata (Ararat region, Armenia) was founded in the early second century BC by the Armenian king Artaxias I, who established it as the capital of his kingdom. Artashat was built on limestone hills rising from the fertile Ararat Valley and quickly became a major metropolis in Armenia, fostering interactions with the Mediterranean, Iran, Mesopotamia, and the northern Caucasus. While historical sources suggest that the area was previously uninhabited, recent archaeological findings indicate that the site had been home to a substantial Urartian settlement during the Iron Age that was possibly abandoned before Artaxias I resettled four centuries later [31–34]. Significant Urartian remains were found on Hill XIII and south of it, complementing the previously known Urartian remains on Hill II, namely a massive Urartian mudbrick wall that was later reused in the Artaxiad fortifications [35,36]. Architecturally, the city employed local construction techniques, with most buildings made of mudbrick placed on low stone foundations [30]. Radiocarbon dating provides clear evidence that both the Urartian and Hellenistic periods of occupation occurred in the same settlement area, and Urartian monuments were apparently reused in the Hellenistic period, such as the Urartian wall on Hill II and the Urartian gate south of Hill XIII. Such a conscious attachment to the Urartian past can also be observed at other Hellenistic sites in Armenia [37] (Fig 2).

The archaeological record preserves the lower courses of some mudbrick walls, although much of the original structure was eroded over time. With this focus, we run an interdisciplinary investigation to study Urartian and Hellenistic mudbrick architecture employing geoarchaeology, archaeobotany, and building archaeology [30]. We took 34 samples from the Urartian defensive wall (Hill II) and buildings from the plain, as well as Hellenistic examples from Hill XIII and the plain, including phases I (ca. 180–50 BC), II (ca. 50 BC-1st half of the 1st century AD), and III (until destruction of the city under Corbulo in 59 AD). The chemical and petrographic data revealed the exploitation of a local source of raw materials for the analyzed earthen building materials. The presence of igneous rocks (e.g., andesite, pyroxenes, pumice, and volcanic glass) is consistent with local outcrops [30:11, 13–14], which resulted from the volcanic activity of Mount Ararat. The presence of polycrystalline extrusive rocks matches the main sources of soil procurement in the north site [30: 21–22], reflecting a strong influence from the igneous activity of the dormant volcano formed during the early Pleistocene.

However, micromorphology and granulometry point to a different structural pattern between Urartian and Hellenistic mudbricks. Urartian mudbricks tend to show a consistently denser structure and the addition of vegetal temper, while Hellenistic mudbricks are characterized by a less dense structure with crudely aligned and rotated voids as evidence of limited tactile manipulation. In addition, the incorporation of EBM aggregates could be recognized in some Hellenistic samples, revealing evidence of Urartian material reuse during the Hellenistic period [30].

### Los Villares de la Encarnación (Spain)

**Fig 3:**
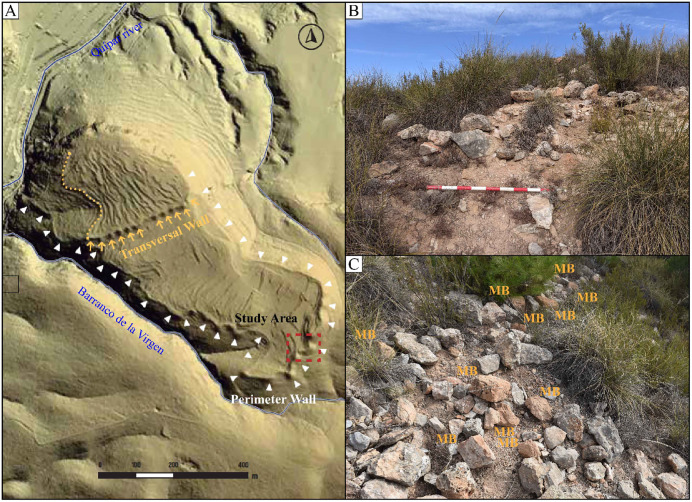
Los Villares de la Encarnación. **(A)** The fortified area of the site, indicated by white arrows. **(B)** Close-up view of the wall. **(C)** Location of the mudbricks and collected samples (Reprinted from [29] under CC BY 4.0 license).

The fortified settlement of Los Villares de la Encarnación (Caravaca de la Cruz, Spain) is an Early Iron Age site located on a hill, rising prominently from the landscape due to its steep slopes of over 40 meters and dominating the surrounding area (Fig 3) [38]. This elevated position gives the settlement extensive control over the basin, including large areas of arable land and pasture between the Argos and Quípar rivers [39]. This location is particularly significant, as it is one of the typical settlements for understanding the development of the inland local communities during the 8^th^ and 6^th^ centuries BC in the face of the impact of Phoenician colonization on the coast.

The archaeological work that has been conducted so far consists of systematic archaeological surveys and material analysis [38,39]. However, we have a rough idea of the structure of the 18 hectares that make up the top of the hill where the settlement is located. Despite the site’s excellent natural defense by escarpments and ravines, the summit was heavily fortified by two sets of ramparts endowed with a significant number of towers or bastions. In its inner space, the survey also points to a certain specialization in some areas dedicated to productive activities, such as pottery manufacture or metallurgy work [39]. The necropolis extends on the plain to the south of the access to the settlement and outside the walls, where several Early Iron Age cremations were located under the Late Iron Age sanctuary of La Encarnación [40].

The use of mudbricks in these autochthonous settlements (so named to define the local communities as opposed to the Phoenician colonies established on the coast) tends to be concentrated in domestic buildings, being limited from the Early Iron Age ramparts [29]. However, we identified the remains of at least one tower built with mudbricks in the southeastern area of the “perimeter” wall [38]. This construction would correspond to a second phase of fortification when the settlement expanded its urban area in a framework of emerging territorial tensions.

With this background, we decided to undertake the geoarchaeological study of the EBMs identified as part of the tower superstructure and expanded our sampling to some nearby structures located inside the walled area that were also built with mudbricks. Extensive descriptions of the recipes and fabrics are included in [29]. We selected 31 samples, 20 from the tower and 11 from the internal structures. The results indicate the existence of different mudbrick recipes, 3 fabrics in total with different subfabrics, according to the chemical and petrographic composition, and the indistinct use of these EBMs with different provenances in the tower and the interior structures [29]. This complexity highlights the adoption of new architectural practices among Early Iron Age communities to build more imposing towers to improve their defensive capacities and might reflect a stronger political image.

## Materials and methods

### Sample selection and image acquisition

For this study, we analyzed a total of 45 thin section samples to validate automated grain morphometry for mudbrick fabric characterization: 27 samples from Artaxata (Armenia) and 18 from Los Villares de la Encarnación (Spain). These samples were previously studied [29,30], providing a well-characterized assemblage with documented compositional variability. The samples selected for the preparation of the thin sections were taken from the fractured areas at the inner part of the mudbricks, avoiding possible post-depositional contamination, and with an orientation perpendicular to the horizontal axis of each mudbrick.

EBM thin sections were previously prepared with an epoxy resin impregnation, mounted and sectioned with a Buehler Petro fine system (Buehler) and finished by hand with silicon carbide to a thickness of 30 µm. The images were acquired using a Leica 2700P DM polarized light microscope equipped with a Flexcam C3 digital camera from the Ceramics and Composite Materials Research Group of the NCSR Demokritos (Athens, Greece).

For each thin section, one representative field of view was documented using a 2.5x objective under cross-polarized (XPL), maintaining constant illumination and exposure parameters. This magnification provides an optimal field of view for representative sampling of groundmass and aplastic inclusions while maintaining sufficient resolution for individual grain characterization. This design tests whether morphometric measurements derived from standard petrographic images can discriminate between assemblages, and it ensures direct comparability with conventional petrographic descriptions, which typically rely on representative fields of observations rather than exhaustive measurements of the entire thin section.

All the images used in this study are in RGB.tif format at 2280 x 2160 pixel resolution, with a spatial resolution of 890 pixels = 1 mm scale factor: 0.001124 mm/pixel = 1.124 µm/pixel. We standardized all acquisition parameters (magnification, illumination intensity, and exposure time) across samples to ensure consistency and comparability of automated measurements [41].

### Image processing and measurements

We employed K-means clustering, an unsupervised machine learning that partitions image pixels into discrete groups based on RGB color similarity [42,43]. For the analysis, we specified three clusters that represent (1) voids, (2) grains (mineral and rock inclusions), and (3) clay matrix. Under XPL, voids typically exhibit light extinction (appear black), grains display variable birefringence colors, and the clay matrix tends to show relatively homogeneous low-order interference colors. We identified the grain cluster per image based on a combination of brightness and chromatic properties, typically intermediate brightness relative to voids and matrix, and verified by visual inspections. The selected cluster was extracted as a binary mask for the morphometric analysis.

We selected K-means clustering over alternative segmentation methods (e.g., U-Net [44] and SegmentAllGrains [45]), following preliminary testing. Our choice was based on the segmentation performance, transparency, and low computational complexity. K-means provides all of the above while requiring substantially less computer resources and eliminating the need for human training data annotations, as well as higher interpretability, both of which are prerequisites for transparent and replicable archaeological investigation.

We refined the binary masks using morphological post-processing to improve grain continuity and reduce segmentation artifacts. Morphological closing (3 iterations, 3 × 3 kernel) was first applied to merge fragmented segments belonging to individual grains. Hole filling was then performed to correct internal voids misclassified as matrix, followed by small object removal (< 100 pixels) to eliminate noise. Finally, we removed the grains touching image borders to avoid truncation bias in morphometric measurements [46].

We assessed the segmentation quality by visually inspecting all masks to identify systematic and random errors. In addition, we evaluated the segmentation robustness by repeating K-means on multiple random initialization seeds (42, 123, 456, 789, and 1011) on a balanced subset of 10 images, 5 for each site. Because K-means cluster labels can permute between runs, grain cluster assignments were matched to the reference segmentation (seed 42) using Dice similarity for label switching. We quantified the agreement across runs using Dice coefficients and Intersection over Union (IoU), which measure the spatial overlap between binary segmentation masks, with values closer to 1 indicating greater agreement between segments [47]. This seed-based stability assessment prioritizes consistency across algorithmic runs, which is crucial for assemblage-level comparison. In addition, this approach follows common practice in unsupervised segmentation, where ground truth data are not available or impractical and where stability in initialization is used to assess reproducibility [48,49].

### Morphometric measurements

We converted all pixel-based measurements to metric units using the calibration factor of 0.001124 mm/pixel (890 pixels = 1 mm), following the established standards for the morphometric measurements for ceramics [11,14] and soils [50–52].

Per-grain measurements included both size and shape metrics. For size characterization, we extracted Feret diameter, minimum Feret diameter, area (in mm), and perimeter. For shape characterization, we calculated circularity, elongation, and aspect ratio.

For each of the 45 images, we calculated population-level statistics to characterize fabric composition. Central tendency measures for grain size included mean, median, and mode Feret diameter (mm). Sorting characteristics were quantified using standard deviation (SD) of Feret diameter (mm) as the primary sorting metric and coefficient of variation (CV) as a normalized sorting measure following Quinn [11]. We assessed the distribution shape through skewness and kurtosis. We quantified the area coverage of the grains as percent area coverage, along with the total number of grains detected per image (S3 File; Tables S1 and S2). Shape characteristics at the population level were represented by mean and standard deviation values for circularity, elongation, and aspect ratio. These aggregated measurements provide a comprehensive morphometric profile for each sample, enabling quantitative fabric characterization and inter-site comparison.

### Statistical analysis and predictive modelling

We employed three complementary approaches to compare Artaxata’s and Los Villares’s morphometric data using univariate tests, multivariate explanatory analysis, and multivariate predictive modeling. Each sample is represented by a single standardized field of view; therefore, statistical comparison and classification are based on image-derived morphometric summaries per sample.

For the univariate comparisons, we used Mann–Whitney U tests [53], a non-parametric test, to assess the significance of morphometric differences between sites for each of ten image-level morphometric features: mean Feret diameter, median Feret diameter, standard deviation of Feret diameter (SD Feret), coefficient of variation of Feret diameter (CV Feret), skewness, kurtosis, percent area coverage, mean circularity, mean elongation, and mean aspect ratio. These ten features, indicated with an asterisk in S3 File; Tables S1 and S2, constitute the core dataset used in all subsequent statistical analyses.

Mann–Whitney tests were selected due to unequal sample sizes (n = 27 vs. n = 18), non-normal distributions observed for several variables, and robustness to outliers commonly present in morphometric data. For each variable, we calculated the U statistic and two-tailed p-value (α = 0.05), along with rank-biserial correlation (r) as an effect size measure, where |r| > 0.5 indicates a large effect [54].

For the multivariate explanatory analysis, we performed Principal Component Analysis (PCA) on the same ten standardized morphometric features to identify dominant patterns of variation and visualize multivariate structure. Prior to PCA, all features were standardized to ensure equal weighting across different measurement scales. We extracted principal components (PCs) accounting for ≥80% cumulative variance and examined the variance explained by each PC, the feature loadings for interpretation of PC axes, and the PC scores for visualization of sample distribution in morphometric space.

To assess whether morphometric measurements could reliably predict site origin, we performed Linear Discriminant Analysis (LDA) using the first principal components as input variables rather than the raw morphometric features, thereby reducing dimensionality while retaining discriminatory information [55]. Model performance was evaluated using Leave-One-Out Cross-Validation (LOOCV), in which each sample is classified using a model trained on all remaining samples (n − 1), providing an unbiased estimate of classification accuracy for small datasets [56]. Classification performance was assessed using overall accuracy, confusion matrices, Receiver Operating Characteristic (ROC) curves, and site-specific precision, recall, and F1-scores.

All computational analysis, including image processing, statistical analysis, and predictive modelling, was implemented in Python 3.12 in Google Collaboratory [57] using skimage [58], scipy [59], and sklearn [60] libraries.

## Results

K-means clustering successfully segmented the thin section images into three distinct material phases based on color and textural similarity under cross-polarized light. The resulting clusters correspond to (1) regions with very low brightness, appearing predominantly black due to light extinction and representing pore space and voids; (2) grains (minerals and rock fragments), characterized by a wide range of birefringence colors from first-order grays to higher-order interference colors; and (3) the clay matrix and groundmass, which display lower overall brightness and more homogeneous coloration (S3 File; Fig S1).

We selected the cluster representing grains for subsequent morphometric analysis. This cluster is distinguished by intermediate median brightness and high chromatic variability, allowing it to be reliably separated from both the darker void cluster and the more uniform clay matrix cluster.

Visual inspection of the segmentation results across all 45 images confirms consistent cluster assignment and generally accurate delineation of grain boundaries. Grains with sharp optical boundaries and strong birefringence, such as quartz grains and limestone fragments, are consistently and accurately segmented across the dataset. Grains that appeared as single entities in the original images but were fragmented during segmentation were subsequently merged through morphological post-processing (closing and grain filling), effectively correcting internal segmentation artifacts and restoring grain integrity for morphometric measurement.

**Fig 4:**
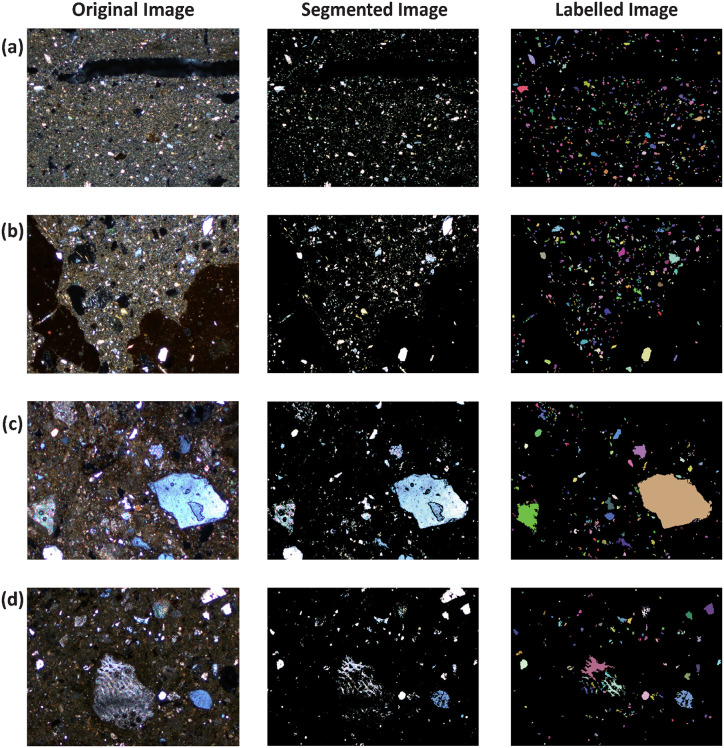
K-means grain segmentation workflow. Each row shows the original thin-section image under cross-polarized light (XPL; left), the binary segmentation of aplastic inclusions obtained by K-means clustering (center), and the labeled grains after morphological processing and individual grain identification (right), for Artaxata samples **(a–b)** and Los Villares samples **(c–d)**. Red arrows in the original images indicate representative aplastic inclusions identified by petrographic observation. Red dashed circles highlight segmentation limitations, including undersegmentation of dark lithologies (e.g., basalt in b) and oversegmentation of fine-grained silt or internally heterogeneous grains (c–d). In the labeled images, colors are assigned randomly to individual grains for visualization purposes only and do not represent mineralogy, size, or morphometric properties (Reprinted and adapted from [29–30] under CC BY 4.0 license).

However, we observe several segmentation challenges for specific mineralogical components that are common to both sites (Fig 4 and S3 File; Fig S2). Basalt grains are frequently excluded from the aplastic inclusion cluster, as their overall brightness approaches that of the void cluster, resulting in their undersegmentation. Similar issues are observed for biocalcarenite grains, which commonly exhibit fragmented or under-segmented representations relative to the original grain outlines. In addition, some calcite grains are over-segmented and partially misclassified within the groundmass cluster. Dice and IoU metrics for segmentation reproducibility show near-perfect results (Dice median = 0.99, minimum = 0.98), with area fraction variability consistently below 1.5%. Detailed robustness analyses are provided in S3 File; Table S4 and Fig S4.

Following the segmentation and image morphological refinement, automated morphometric analysis detected and measured 13818 individual grains across 45 thin section images (mean grains/image: 307): 10021 grains from Artaxata (n = 27 images, 358 mean grains/image) and 3797 from Los Villares de la Encarnación (n = 18, 211 mean grains/image). For each image and site, we calculated population-level statistics for grain size, sorting, shape, and area coverage metrics, and they are presented in the S1 and S2Files.

**Fig 5:**
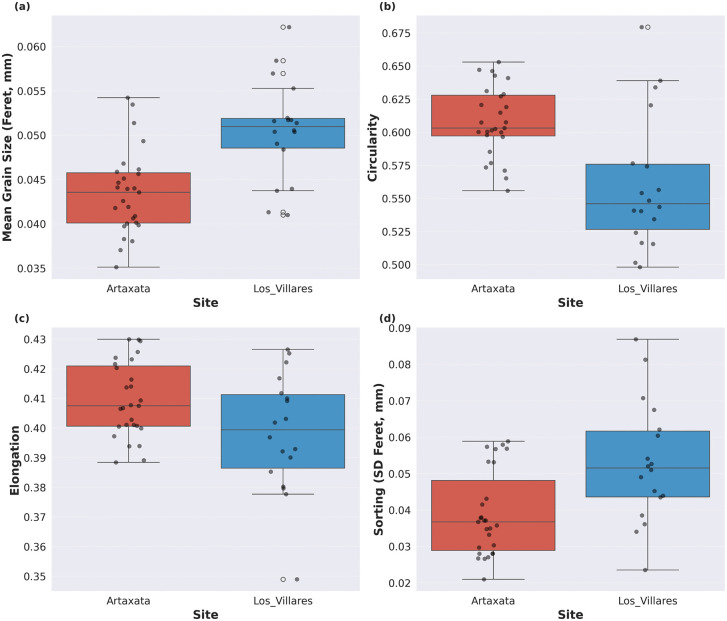
Grain morphometric comparison between sites. Boxplots with overlaid points show **(a)** mean grain size (Feret diameter, mm), **(b)** circularity (1 = circular, lower = irregular), **(c)** elongation (1 = highly elongated, 0 = equidimensional), and **(d)** sorting measured as standard deviation of Feret diameter (higher values = poorly sorted/variable grain sizes, lower values = well sorted/uniform grain sizes) for Artaxata (n = 27) and Los Villares (n = 18). Boxplots display median (center line), interquartile range (box), and range (whiskers).

These measurements reveal systematic morphometric differences between assemblages (Fig 5). Average grain size for Artaxata samples is 0.043 ± 0.004 mm (mean ± SD) in mean Feret diameter, with individual sample values ranging from 0.035 to 0.054 mm. Los Villares samples show larger mean grain sizes (0.050 ± 0.006 mm) and a wider range (0.041 to 0.062 mm), indicating both larger typical grain sizes and greater inter-sample variability.

Sorting characteristics, quantified by the standard deviation of Feret’s diameter, similarly distinguish these sites. Artaxata samples exhibit a mean SD of 0.037 ± 0.009 mm, while Los Villares de la Encarnación shows higher variability (0.052 ± 0.015 mm). Together with coefficients of variation of 82.2% for Artaxata and 94.5% for Los Villares de la Encarnación, it indicates moderately to poorly sorted samples at both sites (Fig 5d). Shape characteristics show each site’s distinct morphometric signatures, with Artaxata grains averaging 0.60 ± 0.03 in circularity and 0.40 ± 0.01 in elongation, indicating moderately rounded to sub-angular grains with low elongation. Los Villares de la Encarnación, on the contrary, are more irregular (circularity 0.54 ± 0.04) and slightly less elongated (0.39 ± 0.02) (Fig 5a and 5b).

Statistical testing confirms the significance of these observed differences. Mann-Whitney U tests identify seven of ten variables as significantly different with large effect sizes (Table 1). Size metrics show highly significant differences: mean Feret diameter (U = 87, p = 0.0003, r = 0.64), median Feret diameter (U = 90, p = 0.0004, r = 0.62), and SD Feret diameter (U = 120, p = 0.0045, r = 0.50), with Los Villares exhibiting larger and more variable grain sizes. The coefficient of variation similarly indicates poorer sorting at Los Villares (U = 151, p = 0.034, r = 0.37). Among shape variables, circularity shows strong differentiation (U = 388, p = 0.0008, r = −0.59), with Artaxata grains more circular than Los Villares grains. Aspect ratio shows moderate differences (U = 350, p = 0.014, r = −0.44), while elongation approaches significance (p = 0.073). Skewness, kurtosis, and area coverage exhibit no significant differences.

**Table 1:** Mann-Whitney U-test results in ten morphometric variables between Artaxata and Los Villares.

Variable	Artaxata (n = 27)	Los Villares (n = 18)	Direction	U	p-value	Effect Size (r)
Mean Feret diameter (mm)	0.04	0.05	VIL > AA	87	0.0003***	0.64
Median Feret diameter (mm)	0.03	0.03	VIL > AA	90	0.0004***	0.62
SD Feret diameter (mm)	0.03	0.05	VIL > AA	120	0.0045**	0.50
CV (%)	82.23	94.53	VIL > AA	151	0.0340**	0.37
Skewness	4.20	3.70	ns	254	0.8078	−0.04
Kurtosis	24.09	18.20	ns	279	0.4108	−0.14
Area coverage (%)	5.34	4.45	ns	271	0.5240	−0.11
Circularity	0.60	0.54	AA > VIL	388	0.0008***	−0.59
Elongation	0.40	0.39	ns	321	0.0726	−0.32
Aspect ratio	1.89	1.85	AA > VIL	350	0.0136**	−0.44

Values represent group means for each variable. The Direction column indicates which site shows significantly higher values (AA > VIL = Artaxata higher; VIL > AA = Los Villares higher; ns = no significant differences). Significance levels are denoted by asterisks (* p < 0.05, ** p < 0.01, *** p < 0.001). Effect size (|r|) is reported as rank-biserial correlation, where |r| > 0.5 indicates a large effect.

**Fig 6:**
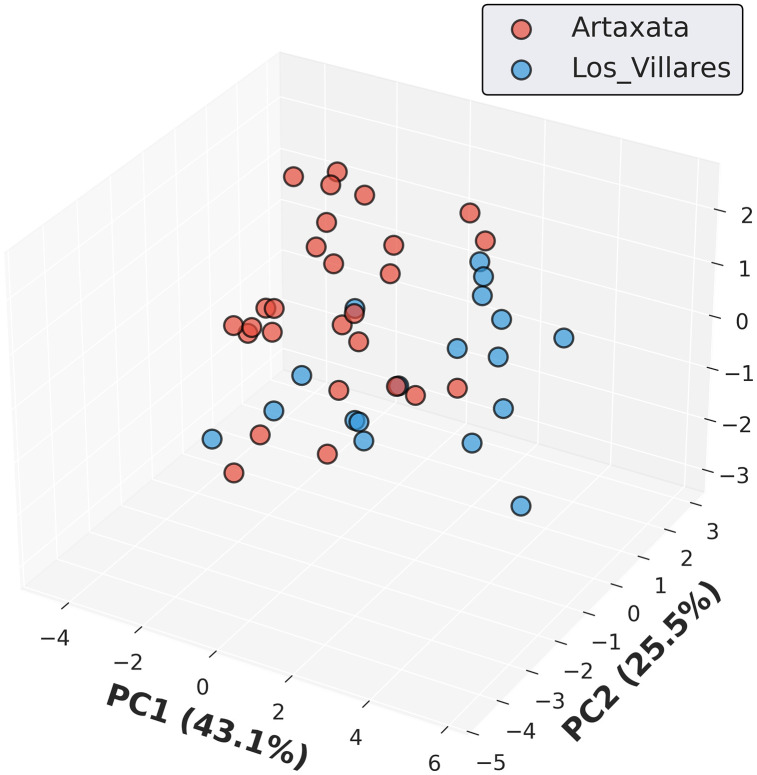
Three-dimensional principal component analysis (PCA) of grain morphometric characteristics. The first three principal components account for 86.1% of total morphometric variance (PC1: 43.1%, PC2: 25.5%, PC3: 17.5%). Each point represents a single thin section sample, with color indicating site origin: Artaxata (red, n = 27) and Los Villares de la Encarnación (blue, n = 18).

Morphometric variation was evident in the PCA. The first three components capture 86.1% of cumulative variance (PC1: 43.1%, PC2: 25.5%, PC3: 17.5%). PC1 loads heavily on size metrics, separating Artaxata samples (negative space: smaller, better-sorted) from Los Villares (positive space: larger, more variable). PC2 captures shape variation (circularity, aspect ratio), while PC3 reflects distribution properties (skewness, kurtosis). In the three-dimensional PC space, Artaxata images cluster tightly in negative PC1 values, whereas Los Villares images occupy positive PC1 and show higher dispersion (Fig 6; PC weights are provided in S3 File; Table S3 along with the cumulative variance, Fig S3).

**Fig 7:**
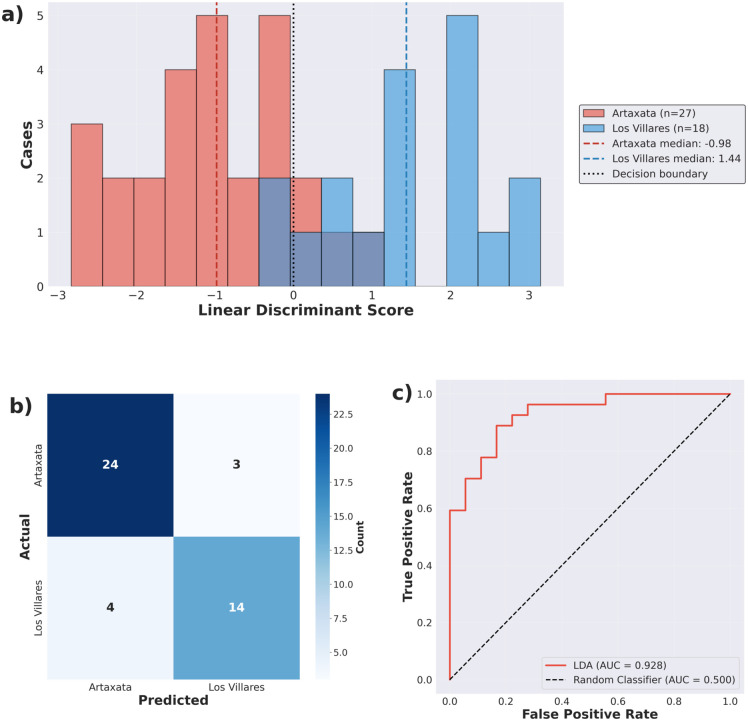
Linear Discriminant Analysis classification performance, evaluated using Leave-One-Out Cross-Validation (LOOCV). **(a)** Distribution of discriminant function scores for Artaxata (red) and Los Villares (blue) samples. Dashed vertical lines indicate median values, demonstrating clear separation in morphometric space with some overlap in the intermediate region. The dashed black line indicates the classification boundary (LD = 0). The overlap near the decision boundary corresponds to the misclassified cases. **(b)** Confusion matrix from LOOCV showing predicted versus actual site assignments. The asymmetric misclassification pattern indicates that Los Villares samples show greater morphometric overlap with Artaxata than vice versa. **(c)** Receiver Operating Characteristic (ROC) curve demonstrating excellent discrimination between sites across all classification thresholds. The curve’s proximity to the top-left corner indicates strong classifier performance, substantially better than random classification (dashed black line).

Building on this multivariate separation, LDA evaluates the discriminatory power of morphometric measurements for site classification (Fig 7). Using the first three principal components as input variables and leave-one-out cross-validation, the model achieves 84.4% accuracy (38/45 samples correctly classified; for Artaxata: AA32_1XP, AA3_2XP, and AA_6_1XP; for Los Villares: VIL5_2XP, VIL14_2XP, VIL23_3XP, and VIL24_5XP). Performance metrics show high precision for both sites (Artaxata: 86.2%, Los Villares: 87.5%), with Artaxata exhibiting higher recall (88%) than Los Villares (77%; Table 2). Misclassification is asymmetric: four Los Villares samples are classified as Artaxata, while only two Artaxata samples are classified as Los Villares (Fig 7b).

**Table 2:** Cross-validated classification performance from Linear Discriminant Analysis (LDA).

Site	Number of images	Precision (%)	Recall (%)	F1-Score (%)	Accuracy (%)
**Artaxata**	27	85	88	87	–
**Los Villares de la Encarnación**	18	82	77	80	–
**Overall**	45	–	–	–	84.4

Metrics are calculated using leave-one-out cross-validation (LOOCV), where each sample is predicted using a model trained on all other samples (n-1). Precision = proportion of samples predicted as a site that were correct; recall = proportion of actual site samples correctly identified; F1-score = harmonic mean of precision and recall. Overall accuracy = 84.4% (38/45 samples correctly classified).

The distribution of discriminant function scores reveals clear morphometric separation between assemblages (Fig 7a). Artaxata samples cluster primarily in negative LD space (median = −0.98), while Los Villares samples occupy positive LD1 space (median = 1.44), with limited overlap around the decision boundary. The overlap region between LD1 ≈ −0.5 and +0.5 contains the six morphometrically ambiguous samples responsible for misclassifications. Los Villares shows more variation along LD1 (a wider distribution) than Artaxata, which has a tighter clustering. The ROC curve demonstrates excellent discrimination across all classification thresholds (AUC = 0.928), confirming robust morphometric separation between assemblages (Fig 7c).

## Discussion

In this study, we evaluated whether automated grain morphometry can be applied reproducibly to archaeological mudbrick thin sections and whether quantitative morphometric analysis could discriminate and capture meaningful differences between assemblages. Our results demonstrate that a transparent and computationally simple image analysis workflow can be successfully applied to earthen building materials, producing consistent grain size, sorting, and shape measurements.

Using these morphometric variables, we identified significant inter-site differences supported by multivariate separation and classification performance. While this study does not produce new archaeological interpretations at this level, it demonstrates that automated grain morphometry provides a robust quantitative framework for comparative analysis of mudbrick fabrics, establishing a new protocol that can be replicated with other sets of EBM thin sections.

The segmentation results demonstrate both the potential and limitations of K-means clustering when applied to mudbrick thin sections. Most aplastic inclusions were consistently segmented across all samples due to their strong birefringence and clear optical properties under cross-polarized light. At the same time, we observed systematic segmentation limitations in both assemblages for specific types of inclusions, particularly basalt, biocalcarenites, and calcite. This under-segmentation pattern is linked to the optical properties of these grains under XPL, where extinction or dark appearance reduces contrast relative to voids or groundmass. Fragmentation and under-segmentation of inclusions such as biocalcarenite further reflect the complexity of internal textures inherent to earthen building materials.

Similar segmentation difficulties for specific mineral phases have been reported in ceramic thin-section studies, where researchers have employed image-specific brightness and contrast adjustments [27] or multistep preprocessing to improve phase discrimination [61,62]. While hybrid approaches combining color and texture-based segmentation or integrating both XPL and PPL images could improve accuracy, such methods often require extensive image-specific parameter tuning. Here, we intentionally adopted a simplified segmentation strategy that prioritizes transparency, reproducibility, and ease of implementation. This choice is a methodological trade-off: rather than increasing segmentation precision for every grain individually, the workflow emphasizes consistency and accessibility, allowing it to be readily applied by petrographers without extensive computational expertise or per-image optimization.

A central outcome of this study is the strong convergence between automated morphometric patterns and previously established petrographic investigations at both Los Villares de la Encarnación and Artaxata. At Los Villares, petrographic analysis identified multiple fabrics and sub-fabrics characterized by wide grain-size ranges, poor sorting, and variable frequencies of coarse inclusions, reflecting flexible raw material selection and preparation practices [29]. These characteristics are mirrored in the morphometric results, which show larger average grain sizes, higher coefficients of variation indicating moderate to poor sorting, more irregular grain shapes, and greater dispersion in multivariate space. The morphometric variability is consistent with the documented existence of at least two geologically distinct raw material procurement identified by Cutillas-Victoria et al. [29], the calcareous marine deposits and the alluvial sediments, each one contributing texturally and mineralogically to the assemblage. Heterogeneous grain size distributions and poor sorting reflect, at least in part, sourcing from geologically heterogeneous catchments rather than a single procurement strategy. However, the prior study also documents deliberate production choices, including the addition of vegetal temper, and the use of different mudbricks from different manufacturing groups within the same structures, suggesting that preparation practices further compounded the natural source variability. The construction of a defensive tower may have required mobilizing materials from multiple sources simultaneously, whether due to supply constraints, the involvement of distinct craft groups operating in parallel, or the demands of a large-scale and potentially rapid construction event.

In contrast, petrographic analysis at Artaxata revealed the dominance of a single fabric with limited internal variability and predominantly sub-angular to sub-rounded grains [30]. This relative homogeneity is reflected in the morphometric results by smaller mean grain sizes, lower dispersion in grain-size distributions, relatively high average circularity values, and tighter clustering in both principal component and discriminant space. The morphometric compactness is consistent with a raw material procurement strategy anchored to the fine-grained alluvial deposits of the Ararat Valley, whose composition is strongly conditioned by volcanic materials from Mount Ararat [30]. These deposits naturally provide silt-dominated, compositionally homogeneous sediment that constrains the range of obtainable grain sizes and shapes. The dominance of a single petrofabric across both Urartian and Hellenistic occupation phases indicates that raw material procurement remained tied to the same geological catchment. Although granulometric analysis documents subtle recipe differences between periods, with Urartian mudbricks showing a more consistent silt fraction and denser structure, Hellenistic mudbricks exhibit a smaller fine fraction and evidence of material reuse [30]. The close correspondence between petrographic fabric homogeneity and morphometric compactness, therefore, likely reflects the single geological availability of Artaxata’s region and a degree of production standardization. It is possible that this standardization reflects the character of Hellenistic Artaxata being a royal city and the Urartian city being subject to centralized palatial organization.

It is important to highlight that automated morphometry does not aim to replicate fabric classification based on mineralogical composition, temper identification, or other micromorphological features. Instead, it isolates fabric variability into quantitative descriptors of grain size, sorting, and shape. The agreement observed at the assemblage level therefore reflects convergence at a specific analytical scale and supports the interpretation of automated morphometric analysis as a complementary analytical tool within EBM studies.

We used a single image per sample derived from the previous petrographic analysis. As such, the morphometric results reflect only specific areas analyzed and are not intended to represent the total variability of the entire mudbrick sample. Rather than generalizing to entire samples, we aimed to test whether morphometric data extracted from standard petrographic images can discriminate fabrics at the assemblage level. We acknowledge that comprehensive intra-sample characterization would require multiple images per thin section or whole-slide imaging; however, this single-image approach is a proof-of-concept for the workflow presented in this study.

In mudbrick assemblages, grain morphometry reflects integrated signals of sediment availability, preparation practices, and degrees of homogenization rather than discrete manufacturing recipes. Grain size and sorting are influenced by both local sedimentary environments and the extent to which materials were selected, mixed, or reworked. Grain shape, on the other hand, reflects source material properties, transport histories, and, in some cases, reuse during mudbrick preparation [1,8,9,11]. Because mudbrick production is inherently flexible and responsive to local conditions, morphometric variability is predicted and archaeologically significant. Quantitative morphometry is thus well adapted for measuring levels of variability and consistency at the assemblage level, particularly in the adaptive manufacture practices of EBMs.

The approach proposed here not only addresses inter-site discrimination but also facilitates the exploration of morphometric diversity in mudbricks across several archaeological scales. Standardized grain morphometric descriptors can be applied to explore diachronic changes in production within a single site, potentially revealing shifts in raw material selection, preparation practices, or labor organization over time. Quantitative morphometry may assist in identifying systematic differences across architectural contexts, such as domestic and non-domestic structures, or between development periods within the same settlement, at the intra-site and intra-regional levels.

The different morphometric fingerprints between Los Villares de la Encarnación and Artaxata may reflect the combined influence of geological availability, production organization, and architectural context, factors that are combined at both sites and cannot be fully disentangled at the current analytical scale. Whether the variability reflects the limits of the available sediments, the logistical demands of a construction at a scale, or the convergence of distinct craft traditions remains an open question that targeted provenance studies and intra-site sampling at higher resolution, including sediment source characterization and expanded thin-section coverage across architectural contexts, would be better placed to address. Automated grain morphometry can serve as a screening and explanatory tool, revealing patterns in technological variability that are difficult to capture just through qualitative description, thus offering a quantitative foundation for comparative investigations across buildings, locations, and regions.

Digital image analysis and machine learning approaches have been widely applied in ceramic petrography, where they have proven effective for quantifying grain-size distributions, mineral inclusions, and temper abundance [10,19,25]. These studies established the methodological foundations for quantitative petrography but focused almost exclusively on fired ceramics. Mudbricks differ fundamentally in heterogeneity, matrix behavior, and technological logic, making direct transfer of analytical protocols inappropriate without explicit validation. By adapting and validating automated grain morphometry for mudbrick thin-section, this study bridges a methodological gap between ceramic analysis and the study of EBM.

The introduction of standardized, reproducible morphometric descriptors has important implications for comparative studies of earthen architecture. Quantitative grain morphometry enables inter-site and inter-regional comparisons that are difficult to achieve using purely descriptive fabric classifications, particularly when dealing with large or heterogeneous datasets. As a screening and comparative tool, morphometry can identify patterns of technological variability and change, guiding more targeted petrographic and micromorphological investigations. By complementing traditional expertise with quantitative measurements, automated morphometry contributes to more transparent, scalable, and systematic analyses of earthen building materials

## Limitations and future perspectives

While the automated workflow successfully distinguishes between assemblages, several methodological considerations inform interpretation and suggest directions for future refinement.

First, image acquisition was limited to a single field of view at 2.5 × magnification per thin section. Although this scale is well suited for fabric-scale petrographic observations, it cannot fully capture within-sample heterogeneity in inherently variable earthen building materials. Future work could integrate larger fields of view or whole-slide imaging approaches (e.g., flatbed scanning) to obtain more representative morphometric characterizations at the sample scale.

Second, image acquisition under cross-polarized light alone imposes specific constraints. While XPL enhances birefringence contrasts and facilitates the identification of many mineral inclusions, it makes it harder to separate certain inclusions, particularly dark lithologies such as basalt, thereby contributing to segmentation errors. Incorporating additional imaging modalities, such as circularly polarized light, which suppresses extinction effects and provides a more uniform contrast between phases, would improve phase discrimination and support a more complete representation of aplastic inclusions.

Third, this study focuses exclusively on quantitative grain morphometry. Although grain size, sorting, and shape were sufficient to discriminate between the two assemblages, morphometry represents only one variable of EBM studies. Future research could include additional variables, including mineralogical composition, matrix texture, and void characteristics, to provide a more holistic reconstruction of raw material selection, preparation, and construction practices. It should be noted that, as a proof-of-concept study based on modest dataset size of two assemblages, the conclusions drawn here support inter-site discrimination but cannot yet support broader generalizations about mudbrick production variability across periods or architectures with different functions. While our results are consistent with established geological contexts [29–30], isolating the specific impacts of raw material versus deliberate technological choices remains a challenge. To address this, future research should combine expanded assemblage coverage with denser intra-site sampling and geochemical analysis. This integrated approach will move the field beyond broad discrimination toward a more nuanced, mechanistic understanding of mudbrick variability.

In terms of segmentation performance, the systematic errors observed are primarily linked to the optical behavior of certain grains and the limitations of RGB-based clustering under XPL illumination. Methodological refinements may include testing alternative or hybrid segmentation approaches and further optimizing post-processing procedures to more effectively address challenging inclusion types. The current dataset contains only two archaeological sites with distinct geological backgrounds. Expanding the reference dataset to include a broader range of regions, chronological contexts, and geological settings will be essential to assess the general applicability and scalability of the proposed workflow.

Regardless of these limitations, the computational approach presented here is intentionally simple and transparent. The workflow can be readily applied to standard thin-section images, provided that acquisition conditions are standardized and cluster assignment is verified, with calibration factors and processing parameters adjusted as needed. As such, it offers a quantitative, standardized, and reproducible complement to established petrographic practices, making it easier for inter-study comparability and more systematic analyses of earthen building materials.

## Conclusion

This study developed and validated an automatic workflow for quantitative grain morphometry in mudbrick thin sections. By using digital image analysis and statistical validation, we created a workflow that automates grain morphometry and uses it as a factor for discriminating two assemblages from two geographically and compositionally distinct sites.

Applied to two assemblages with distinct geological and archaeological contexts, Artaxata (Armenia) and Los Villares de la Encarnación (Spain), this workflow identified systematic morphometric differences between sites. Univariate and multivariate tests show clear separation in morphometric space, and predictive modeling confirms that quantitative morphometry alone can discriminate assemblage origin with high performance.

These results demonstrate that automated grain morphometry provides an objective and scalable complement to established petrographic fabric analysis, supporting inter-study comparability in earthen building material research. Building on computational approaches previously applied to ceramics, this work offers, to our knowledge, the first systematic validation of automated grain morphometry for archaeological mudbricks using thin-section data and statistical classification.

Future research should concentrate on enhancing representativeness and segmentation robustness through the integration of multiple fields of view or whole-slide imaging, the combination of complementary illumination modes (XPL and PPL), and the expansion of quantitative descriptors to encompass matrix and void characteristics in addition to grains. Expanding the reference dataset across regions and time periods will further test the general applicability of the workflow and strengthen its potential as a comparative tool for the study of ancient earthen architecture.

## Supporting information

S1 File Per-grain morphometric measurements. Complete morphometric measurements for all 13,818 individual grains detected across 45 thin section images, including size (Feret diameter, area, perimeter) and shape (circularity, elongation, aspect ratio) parameters.
(CSV)

S2 File Per-image morphometric summary statistics. Population-level statistics were calculated for each of the 45 thin section images, including central tendency measures, sorting parameters, and shape characteristics used in statistical analyses.
(CSV)

S3 File Supplementary tables and figures. Contains per-grain morphometric properties, descriptive statistics, PCA results, K-means segmentation outputs, and robustness metrics for image segmentation.
(DOCX)
