# Supplementary material for: Against the grain: Leveraging machine learning to analyze mudbrick structures
Source: PLoS One. 2026 May 21;21(5):e0349295. doi: 10.1371/journal.pone.0349295 (PMC13193333; doi:10.1371/journal.pone.0349295)
Supplement: S3 File — Contains per-grain morphometric properties, descriptive statistics, PCA results, K-means segmentation outputs, and robustness metrics for image segmentation. (DOCX) [file pone.0349295.s003.docx]

**Table S1. Per-grain morphometric properties extracted from segmented grains in thin-section images.**

| **Property** | **Definition** | **Formula** | **Units** | **Interpretation** |
| --- | --- | --- | --- | --- |
| Feret diameter* | Maximum caliper distance | max(distance between boundary points) | mm | Longest grain dimension |
| Minimum Feret diameter | Minimum calliper distance | min(distance between boundary points) | mm | Shortest grain dimension |
| Area* | grain cros-sectional area | pixel count x calibration factor2 | mm2 | Grain size in mm2 |
| Perimeter | Grain boundary length | boundary pixels x calibration factor | mm | Grain outline |
| Circularity* | Compactness measure | 4π x Area/Perimeter2 | 0-1 | 1= perfect cycle |
| Elongation* | Degree of lengthening | 1 - (minor axis/major axis) | 0-1 | 0 = equant, 1 = linear |
| Aspect ratio* | Length to width ratio | major axis/ minor axis | 1 | 1 =equant, > 2 = elongated |
| Percentage area coverage* | Fraction of images occupied by grains | (Σ grain areas / total image area) x 100 | % | Inclusion proportion within an image |
| Grain count | Number of grains per image | Count of labeled grains | number | Number of grain detected in an image |

Per-grain measurements characterize individual inclusion size and shape. Percent area coverage and grain count are calculated at the image level. All measurements were calibrated using a scale factor of 0.001124 mm/pixel (890 pixels = 1 mm at 2.5× magnification). Grain features marked with an asterisk were used in subsequent statistical testing and multivariate modelling (PCA and LDA).

**Table S2. Descriptive statistics were applied to grain morphometric variables.**

| **Statistic** | **Definition** | **Formula** | **Interpretation** |
| --- | --- | --- | --- |
| Mean | Arithmetic average | x̄ = Σx / n | Average grain size |
| Median | Middle value when data ordered | 50th percentile | Typical grain size |
| Mode | Most frequent occurring value | Peak of distribution | Most common grain size; represents the dominant size fraction |
| Standard deviation* (SD) | Measure of dispersion | SD = √[Σ(x − x̄)² / (n−1)] | Primary sorting metric: higher SD = poorly sorted fabric with wide size range |
| Coefficient of Variation (CV)* | Normalized dispersion | CV = (SD / x̄) × 100 | Normalized sorting independent of mean size; <50% = well sorted, >100% = very poorly sorted |
| Skewness* | Asymmetry distribution | Σ[(x − x̄)³/SD³] / n | Positive = fine-skewed (many small grains, few large); indicates matrix-dominated fabric |
| Kurtosis* | Distribution of heaviness | Σ[(x − x̄)⁴/SD⁴] / n | High values indicate presence of extreme outliers (very coarse or very fine grains) |

These statistical measures characterize the central tendency, dispersion, and distribution shape of grain populations within each thin section image. Statistics were calculated for Feret diameter (size), circularity, elongation, and aspect ratio (shape), where x = individual grain measurement, x̄ = mean, and n = number of grains per image. Variables marked with an asterisk (*) were used in subsequent univariate statistical testing and multivariate analysis (PCA and LDA). We computed these measurements on image-level grain populations calibrated to metric units.

**Table S3. PCA loadings (weights) for the standardized morphometric features of the first three principal components (PC1-3).**

| **Grain Feature** | **PC1** | **PC2** | **PC3** |
| --- | --- | --- | --- |
| **Mean Feret’s diameter** | 0.32 | 0.42 | -0.20 |
| **Median Feret’s diameter** | 0.14 | 0.50 | -0.21 |
| **SD** | 0.46 | 0.05 | -0.14 |
| **CV** | 0.44 | -0.15 | -0.08 |
| **Skewness** | 0.32 | -0.45 | -0.03 |
| **Kurtosis** | 0.29 | -0.47 | -0.05 |
| **Percentage area coverage** | 0.35 | -0.05 | -0.02 |
| **Circularity** | -0.31 | -0.32 | 0.02 |
| **Elongation** | 0.21 | 0.10 | 0.65 |
| **Aspect ratio** | 0.14 | 0.13 | 0.68 |

The loadings correspond to the coefficients of the PCA eigenvectors of the correlation matrix and indicate the contribution of each variable to explain the 86.1% of the total variance.

**Fig S1. K-means clustering output for thin section segmentation.**


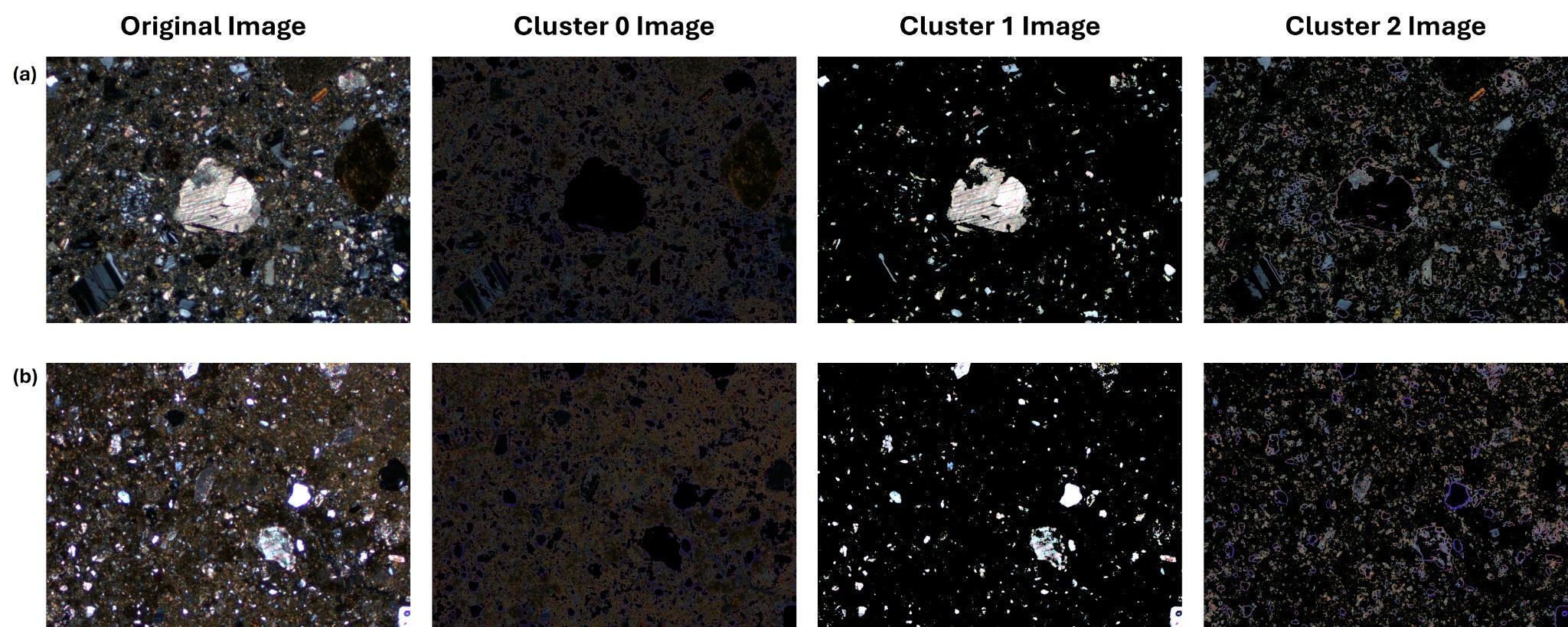


Representative images from (a) Artaxata (AA4_2XP) and (b) Los Villares (VIL23_1XP) showing the original cross-polarized light image and resulting three-cluster segmentation. Cluster 0 represents clay matrix and groundmass, Cluster 1 represents aplastic inclusions (mineral and rock fragments selected for morphometric analysis), and Cluster 2 represents dark regions (voids) and edges from the particles detected. The successful partitioning of material phases based on optical properties under XPL enables automated grain extraction for subsequent morphometric characterization.

**Fig S2. Segmentation workflow and mineralogy-specific challenges.**


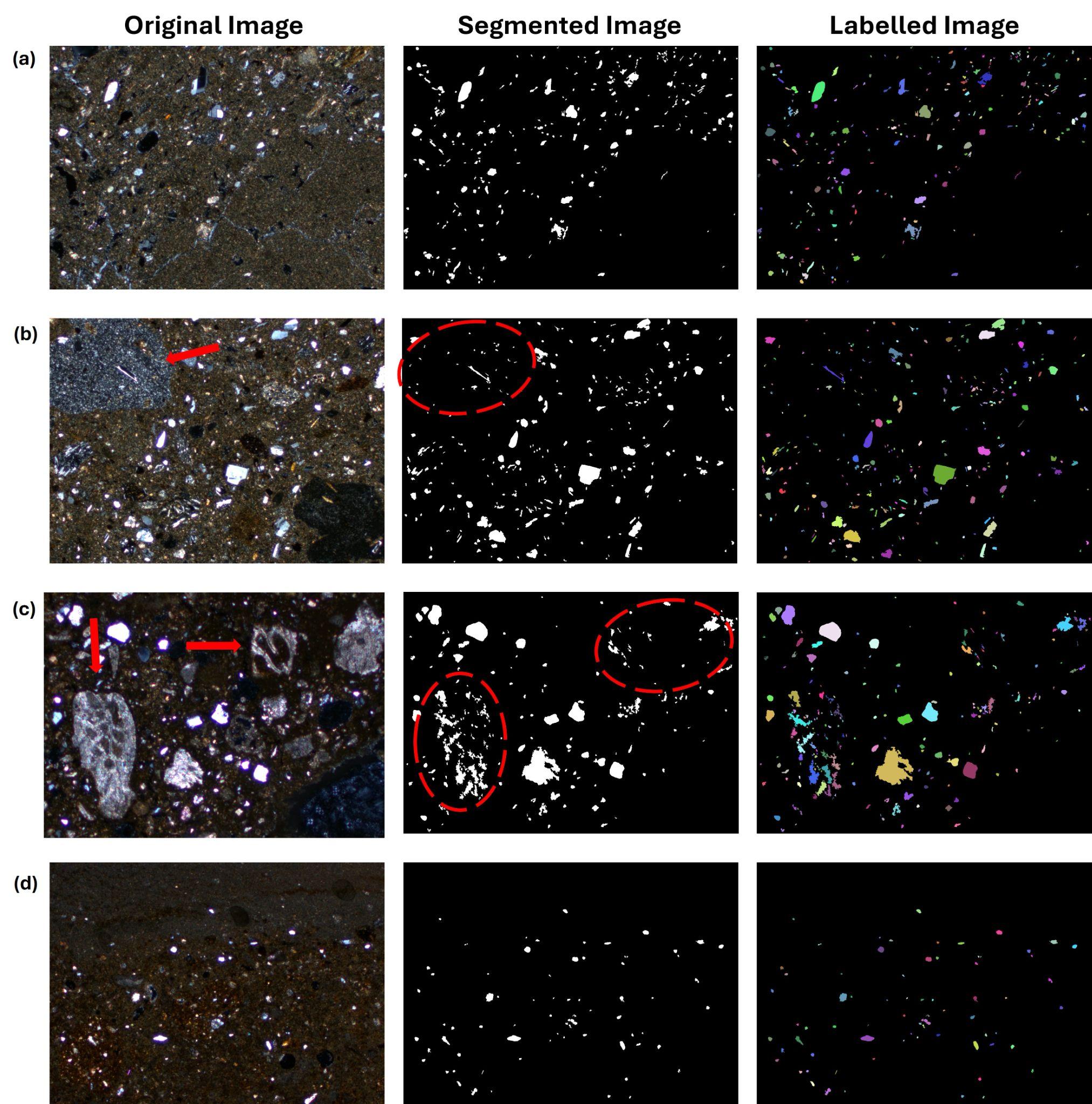


Examples from Artaxata (a-b) and Los Villares (c-d) showing original thin section images (left), binary segmented masks (middle), and color-labeled individual grains (right). (a) AA_13_1XP demonstrates successful segmentation of quartz and feldspar grains. (b) AA21_1XP shows calcite exclusion (red arrow/circle) due to high-order interference colors overlapping with the matrix. (c) VIL_10_2XP illustrates biocalcarenite fragmentation (red arrows/circles) where rock fragments with internal porosity are segmented as multiple objects. (d) VIL_25_4XP represents fine-grained samples with a darker matrix and fewer coarse inclusions.

**Fig S3. Cumulative variance explained by Principal Component Analysis (PCA).**


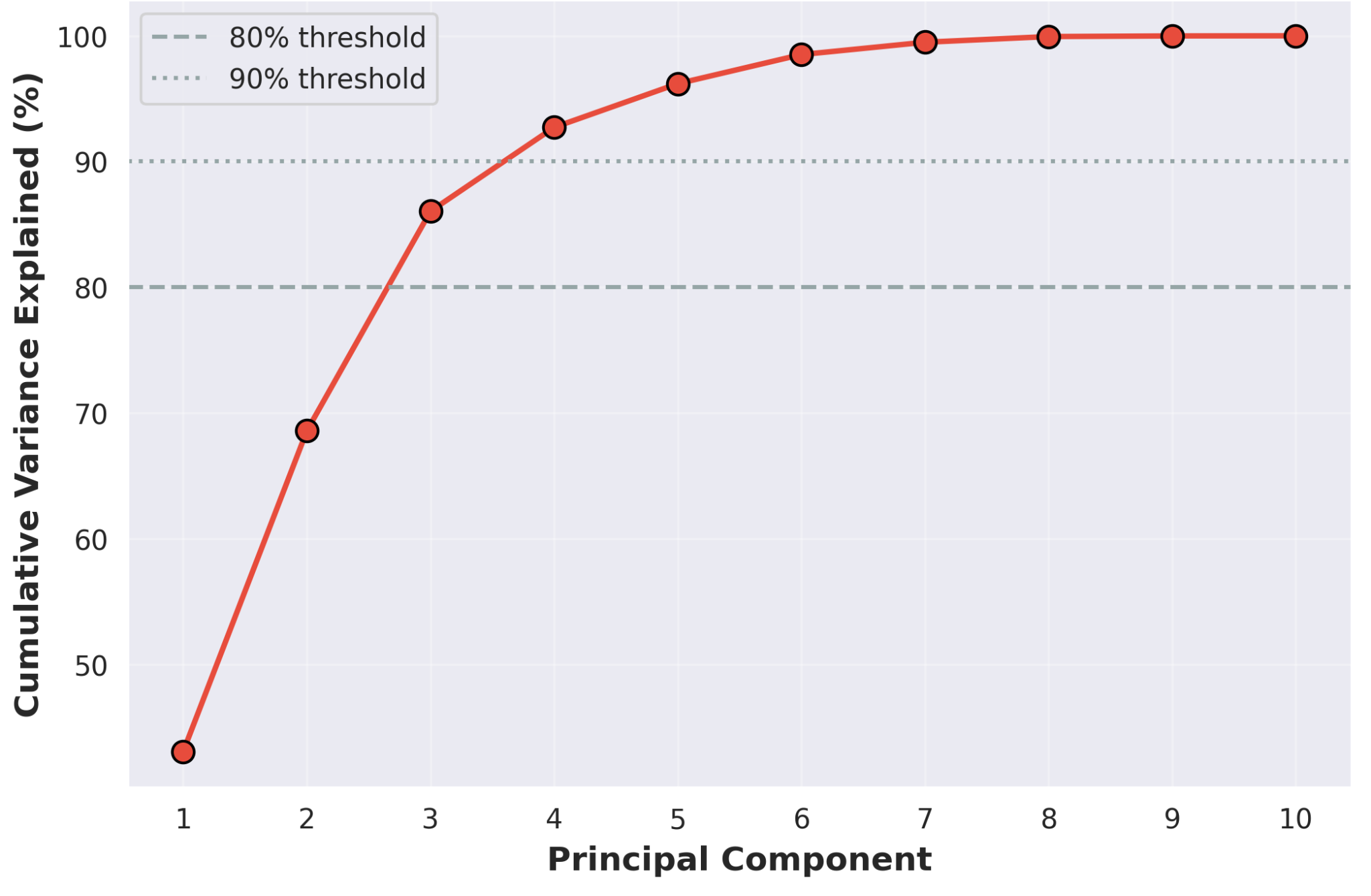


Plot shows the cumulative percentage of morphometric variance captured by successive principal components (PC). PC1 accounts for 43.1% of total variance, PC2 adds 25.5%, and PC3 contributes 17.5%, yielding 86.1% cumulative variance for the first three components (exceeding the conventional 80% threshold, indicated by dashed line). The rapid accumulation of variance in the first three PCs, followed by diminishing returns in subsequent components (PC4-PC10 contributing <14% combined), justified the selection of three principal components as input variables for Linear Discriminant Analysis (LDA). This dimensionality reduction balances comprehensive representation of morphometric variation with overfitting prevention in the small dataset (n=45).

**Table S4. K-means segmentation robustness summary statistics.**

| **Metric** | **Site** | **Mean** | **SD** | **Min** | **Max** |
| --- | --- | --- | --- | --- | --- |
| **Dice coefficient** | Artaxata | 0.998 | 0.004 | 0.987 | 1.0 |
|  | Los Villares | 0.999 | 0.001 | 0.994 | 1.0 |
| **IoU** | Artaxata | 0.995 | 0.007 | 0.975 | 1.0 |
|  | Los Villares | 0.999 | 0.002 | 0.988 | 1.0 |
| **Area fraction CV (%)** | Artaxata | 0.50 | 0.47 | 0.06 | 1.24 |
|  | Los Villares | 0.17 | 0.25 | 0.01 | 0.61 |

Dice coefficient and Intersection over Union (IoU) quantify mask agreement between each random seed (n=10 seeds) and a reference segmentation (seed 42, cluster 1). Area fraction coefficient of variation (CV%) measures stability of grain coverage estimates across seeds. Statistics are based on 50 seed comparisons per site (5 images × 10 seeds).

**Fig S4. Segmentation agreement across random seeds.**


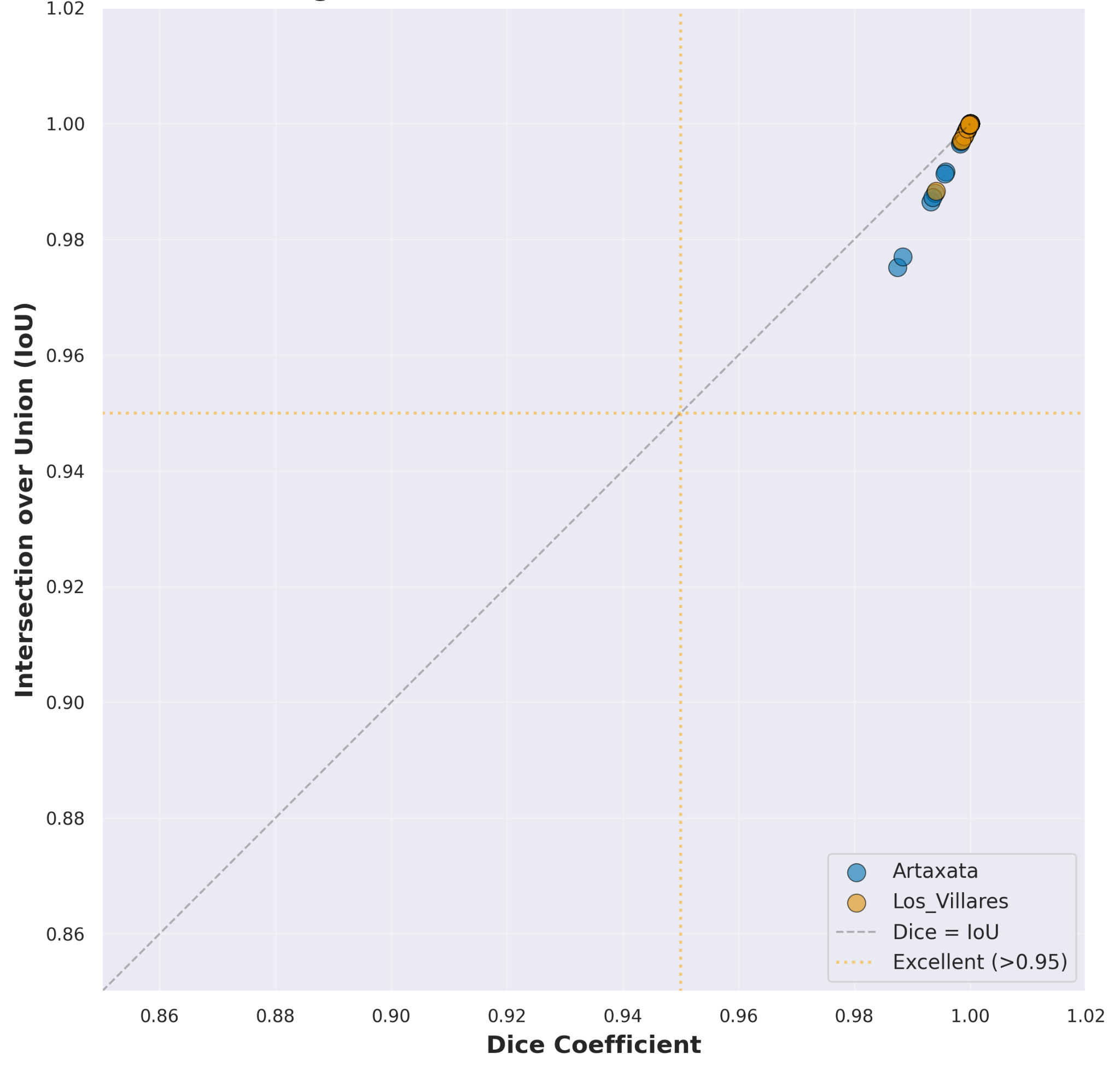


Scatter plot showing the relationship between Dice coefficient and Intersection over Union (IoU) for all image-seed combinations (N=100: 10 images × 10 seeds). Each point represents the agreement between one random seed's segmentation and the reference (seed 42, cluster 1). Tight clustering in the top-right corner (Dice >0.97, IoU >0.97) indicates excellent reproducibility despite random initialization. Los_Villares samples (orange) show slightly higher consistency than Artaxata samples (blue). Dashed lines indicate the diagonal (Dice = IoU) and the 0.95 "excellent agreement" threshold.
